# Cognitive and Mood Effects of a Soluble Mango Leaf Extract (Zynamite^®^ S): A Randomized, Double-Blind, Placebo-Controlled, Crossover Replication Trial

**DOI:** 10.3390/ph19071112

**Published:** 2026-07-18

**Authors:** Ana Beltrán-Arranz, Agustín Aibar-Almazán, David Fuentes-Ríos, Rubén Pérez-Machín, María del Carmen Carcelén-Fraile, Laura López-Ríos, Yolanda Castellote-Caballero

**Affiliations:** 1 Nektium Pharma S.L., Las Mimosas 8, Agüimes, 35118 Las Palmas, Spain; dfuentes@nektium.com (D.F.-R.); rpmachin@nektium.com (R.P.-M.); llopez@nektium.com (L.L.-R.); 2 Department of Health Sciences, Faculty of Health Sciences, University of Jaén, 23071 Jaén, Spain; aaibar@ujaen.es (A.A.-A.); mycastel@ujaen.es (Y.C.-C.); 3 Department of Organic Chemistry, Faculty of Sciences, University of Malaga, Campus de Teatinos s/n, 29071 Málaga, Spain; 4 Department of Educational Sciences, Faculty of Social Sciences, University of Atlántico Medio, 35017 Las Palmas de Gran Canaria, Spain; carmen.carcelen@pdi.atlanticomedio.es

**Keywords:** mango leaf extract, mangiferin, natural nootropic, cognitive performance, mood states, randomized controlled trial, crossover design

## Abstract

**Background/Objectives:** Botanical nootropics are increasingly sought as natural alternatives to synthetic stimulants. *Mangifera indica* leaf extract, standardized to the polyphenol mangiferin, has shown promise in regulating brain activity and enhancing cognitive performance. This study aimed to replicate, in an independent cohort, the acute cognitive benefits of a soluble mango leaf extract (Zynamite^®^ S) following a previous proof-of-concept trial. **Methods:** In a double-blind, randomized, placebo-controlled crossover trial, 88 healthy young adults (aged 18–25) received either a single 100 mg dose of Zynamite^®^ S or a matched placebo. Cognitive performance was assessed using the Trail Making Test (TMT), Digit Symbol Substitution Test (DSST), and Stroop Color-Word Test. Emotional states were assessed via the Profile of Mood States (POMS) at baseline, and then at 30 min, 3 h, and 5 h post-ingestion. **Results:** Zynamite^®^ S supplementation resulted in the replication of previous findings, with significant improvements in mental processing speed, attention, and cognitive flexibility (*p* < 0.05). Notably, this trial identified a rapid onset of action, with significant cognitive and mood improvements detectable as early on as 30 min post-dose and a sustained duration of the effect with benefits observed up to 5 h post-administration. Mood assessments confirmed an overall improvement in emotional balance (*p* < 0.05) by reducing tension, mental fatigue, and low mood during the completion of cognitive-demanding tasks. **Conclusions:** These findings reproduce the acute benefits of Zynamite^®^ S in a healthy young adult population, including a rapid onset of effects and a sustained improvement in cognitive performance and mood over a 5 h window.

## 1. Introduction

The increasing cognitive demands of modern academic and professional environments have spurred a global interest in pharmacological interventions to enhance mental performance [1,2]. While synthetic pharmaceutical agents, such as methylphenidate, modafinil, or piracetam [3,4], are frequently utilized for their potent nootropic effects, their use is often constrained by significant drawbacks [5,6]. These compounds are typically subject to strict regulatory control [7,8] and are frequently associated with adverse effects, including cardiovascular strain, dependency risks, and the well-documented “crash” that follows the peak of their activity [9].

Consequently, research has shifted towards natural, plant-derived nootropics that aim to augment cognitive processes such as memory, attention, and processing speed with a better safety profile [10]. Common examples include *Coffea arabica*, *Bacopa monnieri*, *Panax ginseng*, and *Ginkgo biloba* [11,12]. However, these natural alternatives also present additional limitations. Caffeine sourced from coffee or tea, while being the most widely consumed nootropic worldwide [13], is often linked to jitteriness, increased anxiety, and sleep disturbances [14,15]. Furthermore, other popular plant-derived compounds currently lack extensive scientific evidence and clinical validation required to confirm their nootropic benefits [16]. This gap underscores the necessity for well-designed trials to identify natural ingredients that offer both high efficacy and reliable reproducibility.

Within this search for evidenced-based natural alternatives, *Mangifera indica* (mango) leaf extract has been investigated as a candidate for cognitive enhancement [17]. Historically, various parts of the mango tree have been integral to traditional medicine systems, where leaf decoctions were utilized for their ability to treat fatigue, provide general vitality, and treat a wide range of conditions [18]. This rich ethnobotanical history established the groundwork for modern phytochemical analysis [19,20], which identified mangiferin, a xanthone glycoside, as the primary bioactive. Standardized extracts of this polyphenol are now recognized for their diverse therapeutic potential, including potent antioxidant, anti-inflammatory, and anti-diabetic properties [21].

More recently, its role within the central nervous system (CNS) has been elucidated. While early hypotheses suggested that mangiferin might function as a monoamine oxidase (MAO) inhibitor [22], López-Ríos et al. demonstrated a more specific modulation of the dopaminergic system via the inhibition of catechol-O-methyltransferase (COMT) [17]. By slowing down the degradation of catecholamines in the prefrontal cortex, mangiferin may increase synaptic dopamine levels, thereby facilitating executive control. Following research has demonstrated that a standardized mango leaf extract (Zynamite^®^) containing 60% of mangiferin modulates long-term potentiation (LTP) in the hippocampus and pyramidal neurons [23,24]. Additionally, this extract has been shown to induce brain wave patterns similar to those of caffeine, specifically a reduction in alpha (and theta) wave activity, through electroencephalography (EEG) studies [25].

Initial clinical trials bridged the transition from elucidating the mechanism of action underlying Zynamite^®^ to its clinical efficacy in humans. Preliminary studies demonstrated that an acute 300 mg dose of Zynamite^®^ improved several cognitive domains, including attention, working memory, and psychomotor speed [26]. Despite these encouraging findings from previous studies, the clinical application of mango leaf extracts is limited by mangiferin’s low water solubility and poor bioavailability [27,28], which often results in the requirement for higher dosages to achieve therapeutic effects. To address this challenge, a high-solubility formulation (named Zynamite^®^ S) was developed to increase mangiferin’s absorption profile [29]. A comparative human pharmacokinetic study demonstrated a 3.24-fold increase in mangiferin bioavailability over the first 2 h (AUC0–2h) from Zynamite^®^ S compared to the standard, non-soluble extract in the first two hours after ingestion [29], theoretically allowing for clinical efficacy at significantly lower dosages. A subsequent clinical trial to test whether this enhanced bioavailability allowed for comparable efficacy at a lower dose demonstrated that acute doses of 100 mg and 150 mg of Zynamite^®^ S enhanced both cognitive performance and emotional states [30]. However, in the current landscape of nutritional science, there is a critical need for the replication and validation of such findings to establish a reliable therapeutic threshold and move beyond exploratory results [31,32].

The primary objective of the present study was to perform a confirmatory, double-blind, randomized crossover trial to further examine the acute cognitive and emotional benefits of a 100 mg dose of Zynamite^®^ S. By utilizing a robust study design and cognitive testing battery in an independent cohort, this study seeks to assess whether the previously reported acute effects support Zynamite^®^ S as a fast-acting ingredient for enhancing cognitive performance.

## 2. Results

### 2.1. Sociodemographic and Baseline Characteristics of the Subjects

A total of 88 university students were included in the study from an initial screening of 90 subjects. Study participants were randomized and paired by sex into two groups (placebo or Zynamite^®^ S) that matched similarly in age, gender distribution, physical activity levels, and anthropometric variables, with no significant differences observed between groups. The mean age of the study population was 19.72 ± 1.59 years with a mean BMI of 23.55 ± 3.94 kg/m^2^. A total of 51.1% participants included in the study were female. The demographic and anthropometric data of the 88 subjects included in the study are presented in Table 1 by group.

### 2.2. Cognitive Assessments

#### 2.2.1. Trail Making Test (TMT)

**Figure 1:**
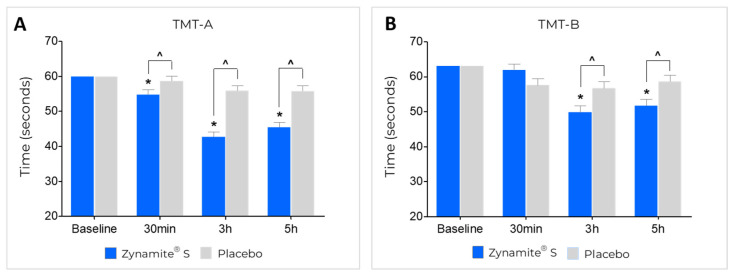
Performance on Trail Making Test (TMT). (**A**) Completion times for TMT-A, representing psychomotor speed. (**B**) Completion times for TMT-B, representing executive flexibility. Data are presented as estimated marginal means (EMM) derived from linear mixed-effects models adjusting for baseline, sequence, and period, with error bars representing the standard error (SE) of the EMM (*n* = 88 per treatment condition). * = *p* < 0.05 difference compared to baseline. ^ = *p* < 0.05 difference between Zynamite^®^ S and placebo.

The TMT assessed different facets of processing speed and executive function, where reduced completion times indicate enhanced performance. In the TMT-A, which evaluates psychomotor speed, the linear mixed-effects model revealed a significant main effect of Treatment (F(1, 433.7) = 63.75, *p* < 0.001), a significant main effect of Time (F(2, 426.2) = 15.97, *p* < 0.001), and, crucially, a significant Time × Treatment interaction (F(2, 426.2) = 5.78, *p* = 0.003), indicating that the temporal trajectory of performance differed between groups in favor of Zynamite^®^ S supplementation. Estimated marginal means (EMM) adjusted for baseline, sequence, and period confirmed sustained improvements in the Zynamite^®^ S group, with significant reductions in completion time relative to placebo at 30 min (Δ = −3.93 s, 95% CI: −7.83 to −0.03, *p* = 0.048), 3 h (Δ = −13.19 s, 95% CI: −17.09 to −9.30, *p* < 0.001), and 5 h (Δ = −10.43 s, 95% CI: −14.33 to −6.53, *p* < 0.001) (Figure 1A). Intragroup analysis in the Zynamite^®^ S group showed significant reductions in completion time at 30 min (*p* = 0.009), 3 h (*p* < 0.001), and 5 h (*p* < 0.001) compared to baseline (Table A1).

In the TMT-B, which assesses executive function and cognitive flexibility, the linear mixed-effects model revealed a significant main effect of Treatment (F(1, 448.1) = 5.08, *p* = 0.025), a significant main effect of Time (F(2, 424.2) = 7.58, *p* < 0.001), and a significant Time × Treatment interaction (F(2, 424.2) = 7.08, *p* < 0.001), indicating a differential pattern of response between treatments over time. Estimated marginal means (adjusted for baseline, sequence, and period) showed that the difference between Zynamite^®^ S and placebo was not significant at 30 min (Δ = +4.17 s, *p* = 0.086) but reached statistical significance at 3 h (Δ = −6.93 s, 95% CI: −11.71 to −2.15, *p* = 0.005) and 5 h (Δ = −6.88 s, 95% CI: −11.66 to −2.10, *p* = 0.005) (Figure 1B). Intragroup analysis showed that Zynamite^®^ S led to progressive improvements from baseline to 3 h and 5 h (both *p* < 0.001), whereas no significant changes were observed within the placebo group (Table A1).

#### 2.2.2. Digit Symbol Substitution Test (DSST)

**Figure 2:**
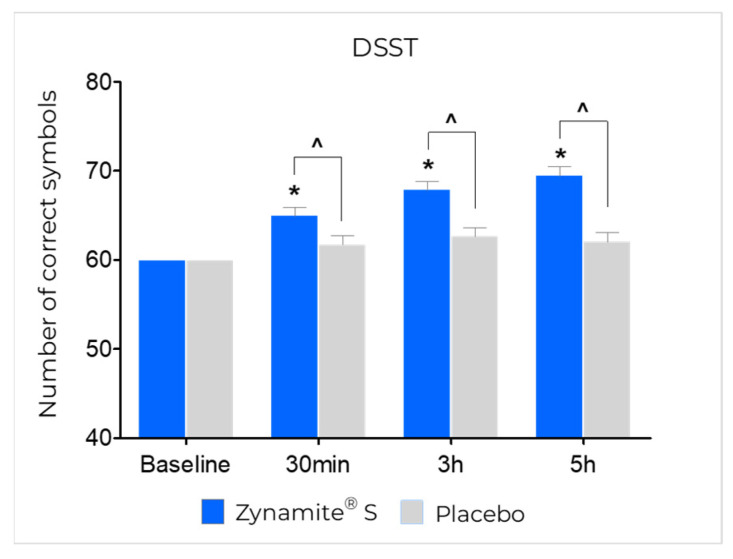
Performance on the Digit Symbol Substitution Test (DSST). Data are presented as estimated marginal means (EMM) derived from linear mixed-effects models adjusting for baseline, sequence, and period, with error bars representing the standard error (SE) of the EMM (*n* = 88 per treatment condition). * = *p* < 0.05 difference compared to baseline. ^ = *p* < 0.05 difference between Zynamite^®^ S and placebo.

The DSST was employed to assess cognitive processing speed, where higher scores represent an increased number of correct substitutions and better performance. The linear mixed-effects model revealed a highly significant main effect of Treatment (F(1, 416.2) = 71.62, *p* < 0.001), a significant main effect of Time (F(2, 413.8) = 5.39, *p* = 0.005), and a significant Time × Treatment interaction (F(2, 413.8) = 3.92, *p* = 0.021), indicating that the trajectory of performance differed between conditions in favor of Zynamite^®^ S. EMM adjusted for baseline, sequence, and period demonstrated a progressive divergence between treatments, with Zynamite^®^ S outperforming placebo at all three post-dose timepoints: 30 min (Δ = +3.21 symbols, 95% CI: 1.09 to 5.32, *p* = 0.003), 3 h (Δ = +5.16 symbols, 95% CI: 3.04 to 7.28, *p* < 0.001), and 5 h (Δ = +7.46 symbols, 95% CI: 5.34 to 9.57, *p* < 0.001) (Figure 2). Furthermore, intragroup analysis revealed that the Zynamite^®^ S intervention led to statistically significant enhancements in processing speed compared to baseline (PRE) at 30 min, 3 h, and 5 h (all *p* < 0.001) (Table A1). Therefore, the effect of Zynamite^®^ S increased progressively across the 5 h assessment window, reflecting sustained enhancement of processing speed.

#### 2.2.3. Stroop Color and Word Test

**Figure 3:**
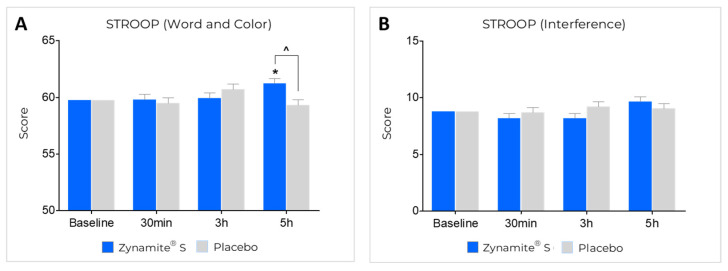
Performance on the Stroop test. (**A**) Stroop Color-Word task scores, where higher values represent better performance under conditions of cognitive conflict. (**B**) Stroop Interference (T) score, where lower values represent better inhibition of cognitive interference. Data are presented as estimated marginal means (EMM) derived from linear mixed-effects models adjusting for baseline, sequence, and period, with error bars representing the standard error (SE) of the EMM (*n* = 88 per treatment condition). * = *p* < 0.05 difference compared to baseline. ^ = *p* < 0.05 difference between Zynamite^®^ S and placebo.

The Stroop Color-Word Test evaluated selective attention and the capacity to inhibit cognitive interference, where higher scores reflect superior performance under conditions of cognitive conflict. Analysis of the Stroop Color-Word task (PC score), which measures the number of correctly identified stimuli under conditions of cognitive conflict, revealed no significant main effects for Treatment (F(1, 449.6) = 1.48, *p* = 0.224) or Time (F(2, 433.6) = 1.36, *p* = 0.258), but a significant Time × Treatment interaction was observed (F(2, 433.6) = 4.26, *p* = 0.015), indicating that the pattern of change over time differed between conditions. EMM adjusted for baseline, sequence, and period showed a significant difference between Zynamite^®^ S and placebo at 5 h (Δ = +1.92 correct responses, 95% CI: 0.62 to 3.22, *p* = 0.004) (Figure 3A). Additionally, a significant performance improvement was observed at 5 h (*p* < 0.001) compared to baseline (Table A1). This pattern reflects a delayed enhancement of cognitive interference inhibition, consistent with the pharmacokinetic profile of mangiferin.

On the other hand, the Stroop Interference (T) score evaluated the capacity to inhibit cognitive interference, with lower values indicating better inhibitory control. The linear mixed-effects model revealed no significant main effects for Treatment (F(1, 519) = 0.72, *p* = 0.398) or Time (F(2, 519) = 2.43, *p* = 0.089), and the Time × Treatment interaction did not reach statistical significance (F(2, 519) = 1.97, *p* = 0.140). Within-group comparisons showed no significant changes from baseline in either treatment arm, and no significant differences between Zynamite^®^ S and placebo were observed at any timepoint (Figure 3B).

### 2.3. Emotional Profile and Mood States

#### 2.3.1. Total Mood Disturbance (TMD)

**Figure 4:**
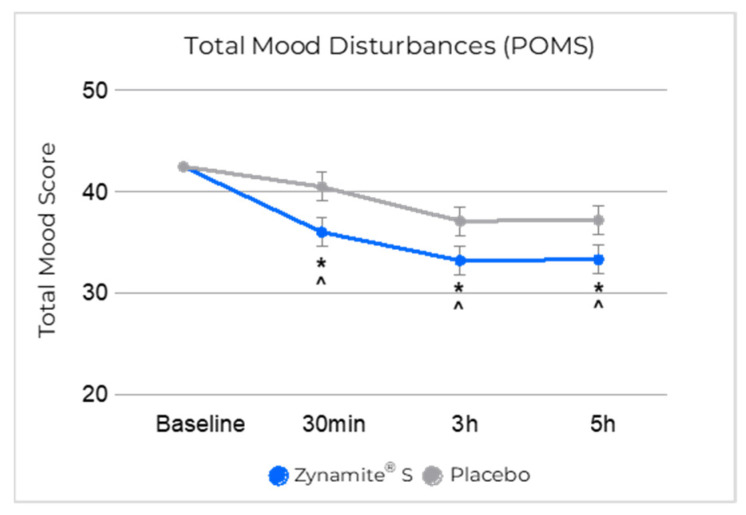
Performance on Total Mood Disturbance (TMD) score. Data are presented as estimated marginal means (EMM) derived from linear mixed-effects models adjusting for baseline, sequence, and period, with error bars representing the standard error (SE) of the EMM (*n* = 88 per treatment condition). * = *p* < 0.05 difference compared to baseline. ^ = *p* < 0.05 difference between Zynamite^®^ S and placebo.

The TMD score provides a composite index of overall affective state, where lower scores reflect more positive mood. The linear mixed-effects model revealed a significant main effect of Treatment (F(1, 447.1) = 25.66, *p* < 0.001) and a significant main effect of Time (F(2, 416.1) = 7.05, *p* < 0.001), while the Time × Treatment interaction was not significant (F(2, 416.1) = 0.05, *p* = 0.954), indicating a consistent treatment advantage across the full assessment window. EMM adjusted for baseline, sequence, and period confirmed that Zynamite^®^ S was associated with significantly lower TMD scores relative to placebo at all three post-dose timepoints: 30 min (Δ = −4.41 points, 95% CI: −7.04 to −1.77, *p* = 0.001), 3 h (Δ = −3.93 points, 95% CI: −6.57 to −1.29, *p* = 0.004), and 5 h (Δ = −3.89 points, 95% CI: −6.53 to −1.26, *p* = 0.004) (Figure 4). Intragroup analysis showed that Zynamite^®^ S exhibited a significant reduction in TMD score from 30 min post-intervention (*p* < 0.001) compared to baseline, an effect that was maintained at 3 h (*p* < 0.001) and 5 h (*p* < 0.001) (Table A2).

#### 2.3.2. Tension Score

**Figure 5:**
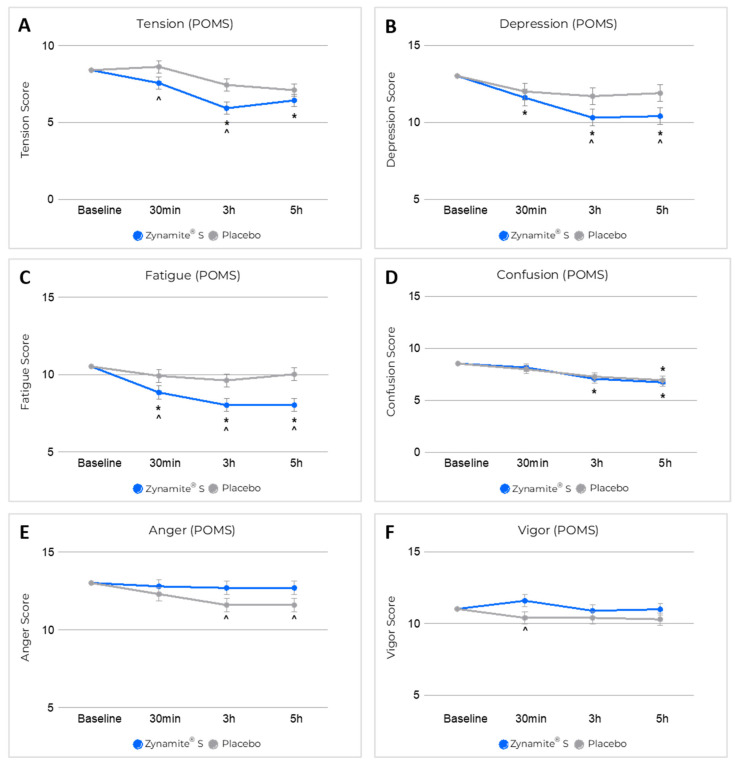
Performance on the six subscales of the Profile of Mood States (POMS): (**A**) Tension, (**B**) Depression, (**C**) Fatigue, (**D**) Confusion, (**E**) Anger, and (**F**) Vigor. Data are presented as estimated marginal means (EMM) derived from linear mixed-effects models adjusting for baseline, sequence, and period, with error bars representing the standard error (SE) of the EMM (*n* = 88 per treatment condition). * = *p* < 0.05 difference compared to baseline. ^ = *p* < 0.05 difference between Zynamite^®^ S and placebo.

The POMS Tension subscale measures somatic tension and anxiety-related arousal, where lower scores indicate reduced tension. The linear mixed-effects model revealed a significant main effect of Treatment (F(1, 419.6) = 15.79, *p* < 0.001) and a significant main effect of Time (F(2, 415.7) = 11.34, *p* < 0.001), with no significant Time × Treatment interaction (F(2, 415.7) = 0.82, *p* = 0.441). EMM adjusted for baseline, sequence, and period confirmed significantly lower tension scores for Zynamite^®^ S relative to placebo at 30 min (Δ = −1.06 points, 95% CI: −1.99 to −0.13, *p* = 0.025) and 3 h (Δ = −1.53 points, 95% CI: −2.45 to −0.60, *p* = 0.001), while the difference at 5 h did not reach statistical significance (Δ = −0.67 points, *p* = 0.155) (Figure 5A). In addition, Zynamite^®^ S intervention demonstrated a rapid and sustained reduction in tension levels compared to baseline at 3 h (*p* < 0.001), and 5 h (*p* < 0.001) (Table A2).

#### 2.3.3. Depression Score

The POMS Depression subscale assesses depressed mood and feelings, where lower scores indicate reduced depressive symptoms. The linear mixed-effects model revealed a significant main effect of Treatment (F(1, 440.8) = 11.25, *p* < 0.001), while the main effect of Time (F(2, 418.1) = 2.55, *p* = 0.079) and the Time × Treatment interaction (F(2, 418.1) = 1.08, *p* = 0.341) were not significant. EMM adjusted for baseline, sequence, and period demonstrated significantly lower depression scores for Zynamite^®^ S relative to placebo at 3 h (Δ = −1.39 points, 95% CI: −2.47 to −0.32, *p* = 0.011) and 5 h (Δ = −1.44 points, 95% CI: −2.51 to −0.36, *p* = 0.009) (Figure 5B). Participants in the Zynamite^®^ S group showed reductions in depression scores relative to baseline across all post-intervention time points (all *p* < 0.001) (Table A2).

#### 2.3.4. Fatigue Score

The POMS Fatigue subscale evaluates subjective feelings of tiredness and exhaustion, where lower scores indicate reduced fatigue. Analysis using the linear mixed-effects model produced a main effect of Treatment (F(1, 457.3) = 29.39, *p* < 0.001), in the absence of significant main Time (F(2, 418.9) = 1.44, *p* = 0.238) or Time × Treatment interaction effects (F(2, 418.9) = 0.94, *p* = 0.390). This pattern points to a stable anti-fatigue effect of Zynamite^®^ S that is maintained throughout the entire assessment window. EMM adjusted for baseline, sequence, and period supported this interpretation, with Zynamite^®^ S yielding consistently lower fatigue scores than placebo at all post-dose timepoints: 30 min (Δ = −1.08 points, 95% CI: −2.02 to −0.14, *p* = 0.025), 3 h (Δ = −1.60 points, 95% CI: −2.54 to −0.66, *p* < 0.001), and 5 h (Δ = −1.99 points, 95% CI: −2.93 to −1.05, *p* < 0.001) (Figure 5C). Zynamite^®^ S intervention significantly reduced fatigue levels at 30 min, 3 h, and 5 h compared to baseline (all *p* < 0.001) (Table A2).

#### 2.3.5. Confusion Score

The POMS Confusion subscale assesses cognitive confusion and disorientation, where lower scores indicate greater mental clarity. The linear mixed-effects model detected no significant main effect of Treatment (F(1, 427.9) = 0.11, *p* = 0.736), and the Time × Treatment interaction likewise failed to reach significance (F(2, 416.6) = 0.25, *p* = 0.778). A significant main effect of Time (F(2, 416.6) = 8.85, *p* < 0.001) reflected a general decline in confusion scores throughout the session. Simple effects analysis adjusted for baseline, sequence, and period confirmed the absence of between-condition differences at any post-dose timepoint (Figure 5D). Within-group paired comparisons revealed significant reductions from baseline in the Zynamite^®^ S group at 3 h and 5 h (both *p* < 0.001), and in the placebo group at 5 h (*p* = 0.01), consistent with the overall time-related decline (Table A2).

#### 2.3.6. Anger Score

The POMS Anger subscale evaluates feelings of irritability, where lower scores indicate reduced anger. The linear mixed-effects model revealed a significant main effect of Treatment (F(1, 444.7) = 8.51, *p* = 0.004), while the main effect of Time (F(2, 416.7) = 0.81, *p* = 0.448) and the Time × Treatment interaction (F(2, 416.7) = 0.54, *p* = 0.582) were not significant. Modest statistically significant differences emerged at 3 h (Δ = +1.15 points, 95% CI: 0.15 to 2.16, *p* = 0.025) and 5 h (Δ = +1.04 points, 95% CI: 0.03 to 2.05, *p* = 0.043), reflecting higher scores in the Zynamite^®^ S group (Figure 5E). Intragroup comparisons showed no significant changes from baseline in either treatment (Table A2).

#### 2.3.7. Vigor Score

The POMS Vigor subscale reflects feelings of energy, enthusiasm, and activity, where higher scores indicate greater vitality. The linear mixed-effects model identified a significant main effect of Treatment (F(1, 427.6) = 8.84, *p* = 0.003), whereas neither the main effect of Time (F(2, 412.9) = 0.66, *p* = 0.515) nor the Time × Treatment interaction (F(2, 412.9) = 0.70, *p* = 0.499) reached statistical significance. Examination of the simple effects adjusted for baseline, sequence, and period revealed that Zynamite^®^ S produced significantly higher vigor scores than placebo at 30 min (Δ = +1.23 points, 95% CI: 0.31 to 2.14, *p* = 0.009) (Figure 5F). Within-group paired comparisons showed no significant changes from baseline in either treatment (Table A2).

## 3. Discussion

The search for natural, non-stimulant cognitive enhancers has led to significant interest in *Mangifera indica* (mango) leaf extract as a consistent modulator of cognitive performance in acute settings [33,34]. This extract is particularly valued for its high concentration of mangiferin, a xanthone glycoside that has demonstrated a unique ability to modulate multiple CNS pathways [35,36,37,38,39]. Recent research studies using a standardized soluble *Mangifera indica* extract (Zynamite^®^ S) to 60% of mangiferin demonstrated enhancement in cognitive performance and improvements in emotional balance [30]. The primary objective of the present study was to determine whether the previously reported cognitive effects could be replicated by replicating the findings of the previous clinical trial regarding the 100 mg dose of Zynamite^®^ S. By utilizing a rigorous double-blind, randomized crossover design in a similar demographic of healthy young adults, this work reproduces the previously reported effects of Zynamite^®^ S as a consistent modulator of cognitive performance in acute settings, specifically confirming the improvements previously observed in executive flexibility and psychomotor speed, while simultaneously improving overall mood states. While exploratory studies often highlight potential benefits, the current work reproduces, in an independent cohort of healthy young adults, the acute cognitive effects previously reported for Zynamite^®^ S. As an internal replication (same research group and facility) rather than an external independent one, these findings speak to the reproducibility of the previously reported acute effects, and a further demonstration of efficacy would require additional independent studies.

### 3.1. Benefits on Cognitive Function

The present study provides further evidence that Zynamite^®^ S acts as a consistent modulator of cognitive performance in acute settings. In line with the previous study, participants in the current trial showed significant reductions in completion times for the TMT. These improvements reflect an enhancement in psychomotor speed and visual scanning (TMT-A), as well as significant gains in cognitive flexibility and executive control (TMT-B) [40,41,42]. Specifically, the improved performance in TMT-B reflects an increased capacity or the ability to switch between mental tasks and a superior efficiency in managing competing information, which requires higher-order cognitive flexibility [43,44]. Similarly, the DSST results in both studies identified a significant increase in the number of correct substitutions, confirming that Zynamite^®^ S supplementation significantly improves the speed of mental processing. Improvement in the DSST indicates a more efficient integration of visual-spatial information and better psychomotor coordination [45,46]. Improvements on this measure have been associated in prior work with better functional abilities and everyday task performance [47].

Quantitatively, baseline-adjusted standardized effect sizes (Cohen’s d) reached medium magnitude for several of the primary cognitive outcomes (TMT-A: d = 0.63 at 3 h and 0.50 at 5 h; DSST: d = 0.40 at 3 h and 0.57 at 5 h; TMT-B: d ≈ 0.30 at 3 h and 5 h), whereas various of the mood effects were small to moderate (Total Mood Disturbance d = 0.21; Fatigue d = 0.39; Depression d = 0.22 at 5h; Tension d = 0.33 at 3 h) (Table A3). These magnitudes indicate that the healthy young adult population was not constrained by a performance ceiling and that the effects are not merely statistically significant but of a magnitude comparable to those reported for established acute cognitive enhancers on the same tasks [48].

A key finding in this replication was that Zynamite^®^ S intake led to significant improvements in TMT performance as early as 30 min for TMT-A and 3 h (for TMT-B) post-ingestion compared to the placebo group, whereas the previous study primarily identified these effects at the 5 h mark. The sustained significance at 3 and 5 h across the TMT as well as DSST batteries suggests that Zynamite^®^ S may offer a “plateau” effect of cognitive enhancement, rather than a sharp peak and crash typically associated with caffeine-based stimulants [48,49]. This increased temporal sensitivity is likely attributable to the higher statistical power of the two-arm design and the adjustment for baseline variance, allowing the rapid-acting pharmacokinetic profile of the soluble formulation to manifest more clearly.

Furthermore, the Stroop Color and Word Test results in this replication provided a more robust confirmation of the selective attention benefits observed. While previous research identified improvements in the interference phase (PC score) exclusively relative to baseline at 5 h, the current findings demonstrated that Zynamite^®^ S significantly outperformed both baseline and the placebo group at this same interval. These improvements in Stroop Color-Word performance reflect an enhancement in selective attention and executive control [50], reinforcing the extract’s role in facilitating cognitive control mechanisms during interference resolution tasks [51].

The improvements observed were measured with well-validated cognitive instruments. While the DSST is widely recognized to correlate with real-world occupational performance and everyday task efficiency [52], the TMT has demonstrated validity across a broad range of neurological and psychiatric conditions and is sensitive to functionally relevant cognitive changes in healthy populations [53]. The present study did not measure academic, occupational, or everyday outcomes directly and, therefore, any translation of the observed acute improvements into real-world benefits remains to be demonstrated.

### 3.2. Benefits on Emotional Balance

The results of the mood assessment suggest that Zynamite^®^ S may contribute to a more stable emotional state during cognitively demanding tasks, in contrast to a traditional stimulant. While both trials identified consistent reductions in TMD, the current study revealed a more immediate shift in the emotional profile. This pattern may help reduce the negative affect associated with prolonged cognitive demand [54]. By mitigating the onset of negative affective states such as Tension and Depression, Zynamite^®^ S intervention appears to foster a resilient mental environment, allowing individuals to maintain a calm-under-pressure during sustained executive focus [55].

Whereas results from TMD, Tension and Depression are consistent with the previous trial, not all mood domains responded uniformly. The emergence of a significant reduction in Fatigue in this replication is particularly noteworthy. In the context of a cognitively demanding environment, fatigue is often a primary barrier to continued performance [56,57]. The fact that participants in this study reported significantly lower fatigue levels indicates that the extract is consistent with preservation of mental energy. Rather than spiking energy levels acutely, as seen with other nootropics like caffeine, Zynamite^®^ S appears to prevent the mental drain associated with repetitive cognitive tasks [58,59,60]. It is worth noting that no active stimulant comparator (e.g., caffeine) was included in the present study, so comparisons with stimulants reflect consistency with the literature rather than direct evidence.

The Vigor subscale followed a different trajectory from the negative-affect domains. Participants reported greater subjective vigor under Zynamite^®^ S than under placebo only at 30 min post-intake. This effect was small (d ≈ 0.15) and did not reflect an increase above their own resting state, indicating that the extract supports mental energy without driving over-arousal. Whereas classical stimulants such as caffeine typically produce a sharp spike in perceived energy followed by a compensatory decline [48], the small vigor signal observed here is more consistent with a state of calm engagement than acute stimulation. Given the exploratory nature of this signal, we interpret it as evidence against over-arousal rather than as enhanced vitality.

The Anger subscale showed a small effect favoring placebo at 3 h and 5 h (*p* = 0.025 and 0.043, respectively). Because the effect was small (d ≈ 0.19) and no within-arm change from baseline was significant in either condition, it is unlikely that these effects reflect a pharmacological action and remain susceptible to residual baseline imbalance.

The Confusion subscale did not replicate the reduction observed in the previous trial, with no significant treatment effect detected (*p* = 0.736). Both groups showed comparable, time-related decreases, suggesting that the improvement in this domain was driven by task familiarization over the session rather than by the intervention.

Altogether, the concurrent reductions in TMD, Tension, Fatigue, and Depression domains, support the characterization of Zynamite^®^ S as a botanical extract that supports mental energy without inducing over-arousal. As POMS was a secondary, exploratory endpoint, these findings remain best regarded as hypothesis-generating.

### 3.3. Mechanistic Basis of Cognitive and Emotional Modulation

The challenge of improving mangiferin’s low solubility and bioavailability has prompted the development of diverse pharmaceutical strategies designed to enhance its systemic uptake. Various delivery systems have been investigated, including molecular encapsulation within natural polymer matrices [61], the use of lipid emulsions containing surfactants [62], and the complexation of the molecule into liposomes or phytosomes [63,64,65,66]. Additionally, structural modifications such as glycosylation [67] and salt formation [68,69] have been explored to improve solubility.

Along these lines, a highly soluble version of mango leaves extract (Zynamite^®^ S) was developed utilizing a controlled ionization process [29]. Comparative pharmacokinetic analysis in human plasma revealed that the Zynamite^®^ S formulation yielded a 3.19-fold increase in mangiferin bioavailability compared to the original non-soluble extract at 1 h post-administration (AUC0–1h); the corresponding increases were 3.24-fold over the first 2 h and 2.44-fold over 24 h [29]. Notably, while peak plasma concentrations of mangiferin are reached at 1–2 h post-ingestion [29], meaningful mangiferin levels are already present at 30 min, consistent with the early mood and moderate cognitive improvements observed at this timepoint. These rapid cognitive and emotional benefits could be explained by an enhanced bioavailability of the soluble formulation, which may allow for faster entry into the circulation than standard, poorly soluble mango leaf extracts. The delayed emergence of full executive enhancement in higher-demand tasks, such as the Stroop Color-Word test, may additionally reflect the progressive accumulation of active metabolites of mangiferin, such as norathyriol [70]. Furthermore, the sustained effects on cognitive enhancement, with performance remaining stable at 3- and 5 h post-ingestion, suggest that mangiferin may offer a steadier influence on neuronal excitability compared to the sharp peaks and subsequent crashes associated with adenosine receptor antagonists like caffeine.

While no enzymatic, neurochemical, or neuroimaging measures were performed in the present study, the following pathways are proposed as hypotheses of potential mechanisms underlying the observed cognitive improvements. Previous studies from our research group and collaborators suggest that Zynamite^®^’s efficacy may involve the selective inhibition of catechol-O-methyltransferase (COMT) [17]. Based on this preclinical evidence, a resulting slowing of catecholamine degradation in the prefrontal cortex could hypothetically increase synaptic dopamine [71,72], consistent with the improvements in processing speed, executive flexibility, and selective attention, given the role of dopamine in the executive control required for the TMT-B and Stroop tasks [73,74]. Complementary preclinical evidence points to additional targets that may contribute to these effects, including acetylcholinesterase (AChE) inhibition [75,76] and BDNF-mediated modulation of synaptic plasticity and long-term potentiation (LTP) [17,25,77]. Although all these mechanisms remain inferential in the present trial, together they suggest that mangiferin may act as a pleiotropic neuromodulator engaging multiple pathways [78].

### 3.4. Limitations and Future Perspectives

While this study demonstrates acute cognitive and mood effects of Zynamite^®^ S in a healthy young adult population, several limitations must be acknowledged to contextualize these findings.

First, the study population was restricted to healthy young adults, a demographic typically characterized by optimal cognitive baseline performance. While this allowed for a clear assessment of the extract’s performance-enhancing potential under controlled conditions, the generalizability of these findings to older populations or individuals experiencing age-related cognitive decline cannot be assumed [79]. Beyond age, all participants were university students from a single academic institution in Spain, representing a homogenous population in terms of culture and geography. Although the use of a homogeneous, healthy young adult population was a deliberate methodological choice to minimize confounding variables and represents the primary intended target population for this product, the benefits observed are limited to this sample. Future studies in more diverse cohorts are planned to establish the broader applicability of the findings, and translation to real-world academic or occupational performance requires ecological studies.

Second, this trial focused exclusively on the acute, single-dose effects of the formulation. Although we observed a sustained “plateau” of cognitive benefit over 5 h, it remains unclear whether chronic supplementation would lead to cumulative gains or the potential development of tolerance. Future longitudinal studies are warranted to explore the long-term safety and efficacy profile of continued use in humans.

Third, several limitations should be noted regarding study design. While the fully counterbalanced crossover design and the dedicated Day 0 familiarization session were implemented to minimize practice effects, residual within-session learning effects cannot be entirely excluded. Next studies should consider the use of parallel alternate test forms at each repetition to further address this concern. Also, although the trial was double-blind and treatments were provided in identical coded opaque capsules, no formal test of blinding efficacy was administered. In addition, the omission of the RAVLT memory battery relative to the previous protocol is noted as a design difference.

Finally, while our behavioral results are consistent with a modulation of dopaminergic and COMT-related pathways, these remain inferential mechanistic hypotheses that are not validated in the present trial and await direct experimental confirmation in humans using neurochemical, enzymatic, or neuroimaging methodologies. In particular, the use of neuroimaging techniques, such as functional Magnetic Resonance Imaging (fMRI) or EEG, would be key to explore the neural mechanisms underlying mangiferin’s rapid onset of action and provide into how the soluble formulation modulates brain activity patterns in real-time [80,81]. These approaches are identified as priority methodological tools for future mechanistic investigations of Zynamite^®^ S CNS activity, alongside biomarker-based assessments such as biochemical and enzymatic assays.

## 4. Materials and Methods

### 4.1. Study Design

Following the initial findings regarding the cognitive effects of Zynamite^®^ S, this study was designed as a confirmatory replication of the previous trial [30]. The current clinical trial employed a double-blind, randomized, placebo-controlled, crossover design at the same University Clinical Research facility. The primary objective was to test the reproducibility of the acute effects of a single 100 mg dose of Zynamite^®^ S compared to a placebo. While the previous study investigated both 100 mg and 150 mg doses, this replication focused specifically on the lower dose to confirm its potency and reliability. Assessment of cognitive performance and emotional state occurred at baseline (PRE) and at 30 min (30 min), 3 h (3 h), and 5 h (5 h) post-administration, maintaining the original study’s temporal framework to ensure data comparability. The study protocol was approved by the Ethics Committee of the University of Atlántico Medio (CEI/05-009) on the 9 May 2025, and all endpoints were pre-specified in the ethics-approved protocol prior to enrollment on the 12 May 2025. The trial was subsequently registered at ClinicalTrials.gov (NCT07126717) on the 11 August 2025.

### 4.2. Study Subjects

A total of 88 healthy university students (undergraduate or postgraduate) were included in this replication trial. Initially, 90 students were screened and contacted, but two participants withdrew prior to completion. All participants were aged between 18 and 25 years and were confirmed to be free of any relevant medical conditions or diseases upon enrollment. To maintain consistency with the original validation framework [30], the exclusion criteria remained the same. Individuals were excluded if they met any of the following: (i) presence of chronic diseases (e.g., asthma, Type 1 diabetes, thyroid disorders, or autoimmune conditions); (ii) diagnosed psychiatric conditions, including anxiety, major depression, eating disorders, or Attention-Deficit/Hyperactivity Disorder (ADHD); (iii) pregnancy or lactation; (iv) known hypersensitivity or allergy to the study’s nutritional components; (v) sensory or cognitive barriers such as intestinal malabsorption, dyslexia, or color blindness; or (vi) the use of psychoactive substances or medications. Lifestyle exclusions included the consumption of alcohol or high daily caffeine intake (>500 mg, equivalent to approximately six 150 mL cups of filtered coffee).

### 4.3. Randomization and Blinding

**Figure 6:**
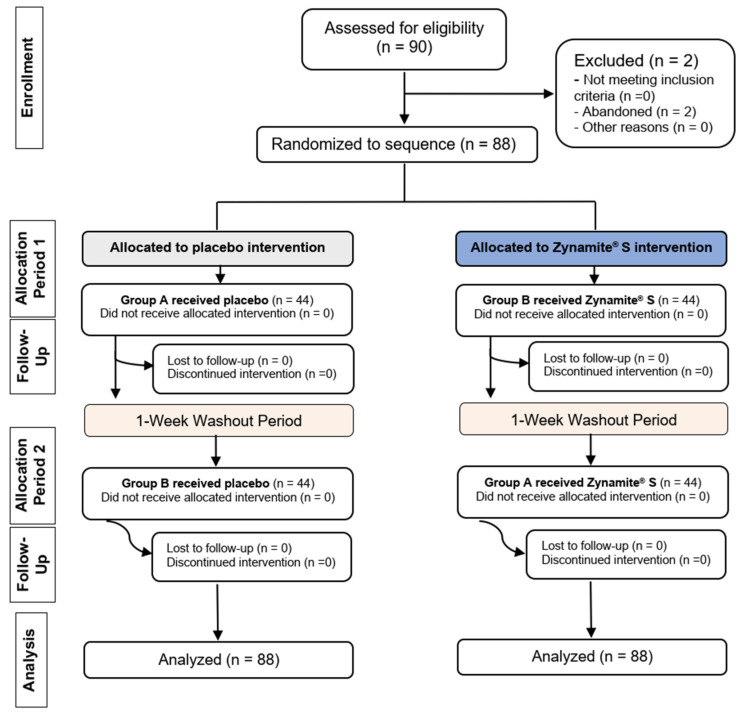
CONSORT flow diagram illustrating the stages of recruitment, eligibility screening, and treatment allocation. The diagram specifies the number of participants included in the final analysis and the attrition rates at each assessment point.

Participants were randomly assigned and matched by sex into two experimental groups (Figure 6). Following a crossover design, Group A received the placebo and Group B received 100 mg of Zynamite^®^ S on the first intervention day. After a 7-day washout period, participants received the alternate treatment. The study remained double-blind; neither the participants nor the researchers involved in data collection were aware of the treatment assignment. The randomization sequence was computer-generated by an independent researcher and statistician not involved in the clinical assessment.

### 4.4. Intervention

Participants received either a single 100 mg dose of soluble mango leaf extract (Zynamite^®^ S, standardized to 60% mangiferin; Nektium Pharma S.L., Agüimes, Spain) or a matched placebo containing maltodextrin. Each treatment was administered in identical, opaque, third-party-coded capsules with a glass of water, ensuring allocation concealment. On each experimental day, capsules were consumed 30 min prior to the first post-dose assessment to allow for initial metabolic uptake. To prevent carryover effects, a 7-day washout period was kept between treatments. The half-life of mangiferin from the Zynamite^®^ S formulation following oral administration in humans is 2.98 ± 2.04 h [29], meaning that complete systemic clearance would be expected within approximately 15 h of the last dose (5 × T_1/2_) [82]. The 7-day washout period provides a conservative safety margin for complete compound elimination.

The manufacturing process and full chemical characterization of the Zynamite^®^ S formulation have been previously described by Fuentes-Ríos et al. 2025 [29]. Briefly, the batch used in this trial was fully characterized under GMP conditions before use. Mangiferin content was 60.0% (*w*/*w*) by HPLC-PDA (Figure S1), as well as polysaccharide, polyphenol, and glycosyl content was determined. On the other hand, safety markers, including urushiols and tertiary alkaloids, were not detected by LC-MS/MS (Figure S2). The complete compositional profile of batch ZYN60S24 is provided in Table S1.

Adverse events and tolerability were formally monitored throughout both experimental sessions (Day 1 and Day 7) using structured reporting forms. No adverse events were observed in any participant across either treatment condition. The safety profile of Zynamite^®^ has been previously established through a comprehensive OECD- and GLP-compliant toxicological evaluation [83]. The no-observed-adverse-effect level (NOAEL) was determined to be 2000 mg/kg bw/day, the highest dose tested. The 100 mg single dose administered in the present trial represents a negligible fraction of this threshold, providing a substantial safety margin.

### 4.5. Procedure

Participants attended the research facility on three occasions: Day 0 (screening and familiarization), followed by two experimental sessions (Day 1 and Day 7) conducted one week apart to ensure a washout period between crossover conditions.

Screening and Familiarization (Day 0): Eligibility was confirmed through informed consent and assessment of inclusion/exclusion criteria. Baseline sociodemographic and clinical metrics such as age, sex, weight, height, and body mass index (BMI) were recorded, and participants were familiarized with the cognitive testing batteries to minimize learning effects.

**Figure 7:**
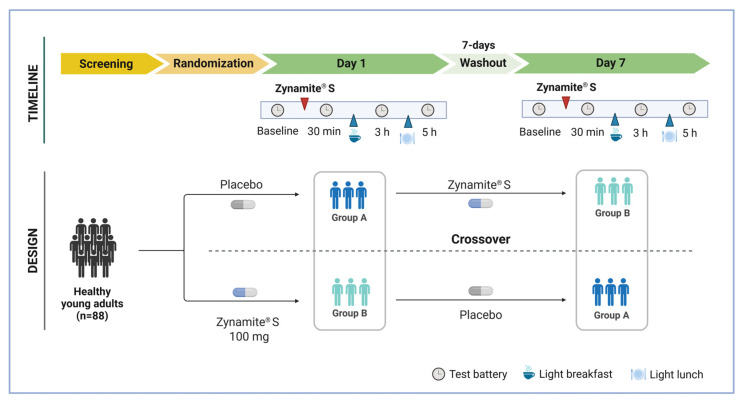
Schematic illustration representing the study design, assessment times, and procedures throughout the experimental sessions. This figure was created in BioRender. López, L. (2026) https://BioRender.com/12s0lef (accessed on 8 July 2026).

Experimental Sessions (Day 1 and Day 7): Participants arrived at the laboratory at a consistent morning hour. Strict pre-test protocols were enforced: 24 h abstinence from alcohol, caffeine restriction from the previous night, and a light breakfast (cereals/toast) consumed at least one hour before arrival. Compliance was verified upon entry. Throughout the test day, participants were restricted to water intake and prohibited from chewing gum or consuming external food. On each visit, cognitive and mood scores were collected 30 min prior to supplementation and 30 min, 3 h and 5 h post-dose. At every assessment, the cognitive and mood tests were administered in the same fixed order across both sessions, so that any order-related fatigue was held constant across conditions. The same test forms were administered at each session, and practice effects were minimized through a Day-0 familiarization session and full counterbalancing and were further controlled by the placebo condition. A light snack (juice and digestive biscuits) was provided after the 30 min assessment, and a standardized lunch (cheese sandwich, chips, and yogurt/custard) was served following the 3 h assessment (Figure 7).

### 4.6. Experimental Outcomes

Cognitive performance and emotional state were evaluated using a battery of validated neuropsychological instruments, consistent with the protocols established in our previous trial [30]. As pre-specified in the ethics-approved study protocol, the Trail Making Test (TMT), Digit Symbol Substitution Test (DSST), and Stroop Test were designated as the primary cognitive outcomes of this study. The Profile of Mood States (POMS) and its subscales were pre-specified as secondary outcomes.

#### 4.6.1. Cognitive Function

TMT: This test assessed psychomotor speed and executive control. Part A (TMT-A) measured visual-motor tracking and speed (connecting numbered circles), while Part B (TMT-B) evaluated cognitive flexibility and executive function (alternating between numbers and letters) [53,84]. Performance was quantified by the time taken (seconds) to complete each part, where shorter durations indicated superior performance.

DSST: A paper-and-pencil task used to measure processing speed. Participants matched specific symbols to numbers according to a provided key [52]. The final score was the total number of correct substitutions completed within 90 s.

Stroop Color-Word Test: This instrument evaluated selective attention and inhibitory control across three 45 s phases: word reading (P), color naming (C), and the interference phase (PC), where ink color and word meaning were incongruent. A standardized Stroop Interference (T) score was calculated, with higher scores reflecting a greater ability to inhibit cognitive interference [85,86].

#### 4.6.2. Mood Assessment

To assess emotional state, participants completed the Spanish version of the POMS 65-item questionnaire [87]. This tool measures six subscales: Tension, Depression, Anger, Fatigue, Vigor, and Confusion. A Total Mood Disturbance (TMD) index was derived by summing the negative subscale scores and subtracting the Vigor score (potential range: −32 to 200). Higher TMD scores indicate increased psychological distress or mood disturbance.

### 4.7. Sample Size Calculation

The sample size was determined a priori using G*Power (version 3.1.9.7). Given the crossover design of the study, in which each participant received both treatments (Zynamite^®^ S and placebo) and was assessed at four time points (baseline, 30 min, 3 h, and 5 h) during each intervention session, the power analysis indicated that a minimum of 82 participants was required to detect a medium effect size (f = 0.24) with 80% power (1 − β = 0.80) at a significance level of α = 0.05. The final sample of 88 participants ensured adequate statistical power.

### 4.8. Statistical Analysis

The statistical analysis was designed to align with a double-blind, randomized, two-period crossover clinical trial. All analyses were conducted using Jamovi (version 2.3) with the GAMLj module. Statistical significance was set at α = 0.05.

As pre-specified in the ethics-approved study protocol, three cognitive outcomes were pre-specified as co-primary endpoints assessing functionally distinct cognitive domains: the Trail Making Test (TMT) for visual attention and task switching, the Digit Symbol Substitution Test (DSST) for processing speed, and the Stroop Color-Word Test for selective attention and cognitive inhibition. Each primary endpoint provides independent evidence within its own cognitive domain, rather than serving as a redundant test of the same construct. The Profile of Mood States (POMS) Total Mood Disturbance score and the six individual subscales were pre-specified as secondary endpoints.

The primary treatment effect was evaluated using a linear mixed-effects model (LMM) fitted by Restricted Maximum Likelihood (REML) for each outcome variable. The model included Treatment (Placebo vs. Zynamite^®^ S), Time (30 min, 3 h, 5 h post-dose), Sequence (PZ vs. ZP), and Period (1 vs. 2) as fixed-effect factors, with the Treatment × Time interaction as the primary parameter of interest. The pre-dose value of each session was incorporated as a fixed-effect covariate to account for within-subject baseline variability across all endpoints. A residual imbalance in Day-1 pre-dose baseline scores between treatment sessions was observed for several outcomes (Table A4); because each session’s pre-dose value was included as a covariate, the resulting baseline-adjusted EMMs account for this imbalance in all reported treatment effects. Subject identifier was included as a random intercept to model the within-subject correlation arising from repeated administration of both treatments. Denominator degrees of freedom were estimated using the Satterthwaite approximation.

Period and sequence effects were directly modeled as fixed effects within the LMM, providing simultaneous estimation with appropriate standard errors. The fully counterbalanced design (44 participants per sequence) ensures symmetric distribution of period effects across treatment conditions. Sequence effects were assessed by comparing Day 1 pre-dose baseline scores between sequences using independent-samples *t*-tests, period effects were assessed by comparing Period 1 vs. Period 2 baseline scores within subjects using paired-samples *t*-tests, and carryover effects were assessed by comparing Day 7 pre-dose baseline scores between sequences using independent-samples *t*-tests (Table A4).

Type I error rate was controlled through complementary structural safeguards: pre-registration of endpoints prior to study initiation, precluding post hoc endpoint selection and selective reporting; selection of functionally distinct cognitive domains (in line with FDA guidance on multiple endpoints [88]); and interpretation based on the directional consistency of effects across domains rather than isolated significant findings. Nominal *p*-values are reported for all simple effects, alongside point estimates (Δ) and 95% confidence intervals, allowing effect size interpretation independent of significance thresholds.

Estimated marginal means (EMM) with 95% confidence intervals were derived from the linear mixed-effects model, evaluated at the grand mean of the baseline covariate. Simple effects of Treatment within each Time level were computed to test for differences between Zynamite^®^ S and placebo at each post-dose timepoint (30 min, 3 h, and 5 h).

Standardized effect sizes (Cohen’s d) for the treatment contrast at each timepoint were derived from the baseline-adjusted mean difference divided by the pooled baseline standard deviation, reported with 95% confidence intervals and interpreted using Cohen’s benchmarks (0.2 small, 0.5 medium, 0.8 large). The POMS subscales and the secondary Stroop contrasts were treated as exploratory and reported without correction for multiple comparisons, whereas the three pre-specified cognitive primaries (TMT, DSST, Stroop) remained significant under conservative correction.

Model assumptions (normality and homoscedasticity of residuals) were verified via Shapiro–Wilk and Kolmogorov–Smirnov tests, Q-Q plots, and residual-predicted scatterplots. Model fit was characterized by marginal and conditional R^2^ statistics, with the intraclass correlation coefficient (ICC) reported as an indicator of within-subject correlation strength.

Complementarily, within-group paired *t*-tests were conducted to evaluate temporal changes from baseline within each treatment arm. For each treatment, scores at 30 min, 3 h, and 5 h were compared against the pre-dose baseline using paired-samples *t*-tests. These analyses serve a descriptive purpose to characterize the within-group trajectory over time, independent of between-treatment comparisons, and are reported with nominal *p*-values.

## 5. Conclusions

The present study demonstrates significant acute improvements in processing speed, executive function, and selective attention following a single 100 mg dose of Zynamite^®^ S in healthy young adults, replicating findings from a previous clinical trial. Statistically significant effects were observed across multiple validated cognitive instruments and sustained over a 5 h post-dose window, reproducing the acute cognitive and mood benefits previously reported in this population. These results support the continued investigation of Zynamite^®^ S as a caffeine-free candidate for acute cognitive support, while underscoring the importance of replication in nutritional science.

## Figures and Tables

**Table 1:** Preintervention sociodemographic and clinical characteristics of the participants as a whole and by group.

Variable		Group A	Group B	*p*-Value
Age (years)		19.52 ± 1.53	19.91 ± 1.65	0.259
Sex	Male (%)	21 (47.73)	22 (50.00)	0.766
	Female (%)	23 (52.27)	22 (50.00)	
Sport	Yes (%)	32 (72.73)	31 (70.45)	0.924
	No (%)	12 (27.27)	13 (29.55)	
Height (m)		1.71 ± 0.09	1.72 ± 0.10	0.141
Weight (kg)		68.80 ± 13.80	71.50 ± 18.20	0.314
BMI (kg/m^2^)		23.30 ± 3.27	23.80 ± 4.61	0.174
Waist (cm)		74.60 ± 9.58	77.90 ± 12.90	0.414
Hip (cm)		94.50 ± 10.60	97.70 ± 14.40	0.153
WHR		0.79 ± 0.07	0.80 ± 0.07	0.571

Quantitative variables are presented as mean and standard deviation. Qualitative variables are presented as frequency and percentage. BMI: Body Mass Index. WHR: Waist-to-Hip Ratio.

## Data Availability

The original data presented in this study are openly available in OSF public repository at https://osf.io/c3pnh/overview (accessed on 8 July 2026).

## Supplementary Materials

The following supporting information can be downloaded at https://www.mdpi.com/article/10.3390/ph19071112/s1, Figure S1: HPLC-PDA chromatogram and inline UV spectrum of Zynamite^®^ S (batch ZYN60S24); Figure S2: HPLC-MS/MS chromatographic and mass spectral characterization of Zynamite^®^ S (batch ZYN60S24); Table S1: Phytochemical characterization of Zynamite^®^ S (batch ZYN60S24).

## Abbreviations

The following abbreviations are used in this manuscript:

AChE	Acetylcholinesterase
ADHD	Attention-deficit/hyperactivity disorder
BMI	Body mass index
CNS	Central nervous system
COMT	Catechol-O-methyltransferase
DSST	Digit symbol substitution test
EEG	Electroencephalography
EMM	Estimated marginal means
fMRI	Functional magnetic resonance imaging
ICC	Intraclass correlation coefficient
LMM	Linear mixed-effects model
LTP	Long-term potentiation
MAO	Monoamine oxidase
NOAEL	No Observed Adverse Effect Level
POMS	Profile of mood states
RAVLT	Rey auditory verbal learning test
REML	Restricted Maximum Likelihood
SD	Standard deviation
SE	Standard error
TMD	Total mood disturbance
TMT	Trail making test
TMT-A	Trail making test part A
TMT-B	Trail making test part B
WHR	Waist-to-hip ratio

## Appendix A

**Table A1:** Descriptive statistics for cognitive variables and longitudinal change. Values represent raw means and standard deviation for each assessment. Percentage of change (%) indicates the mean individual variation from baseline to each timepoint. SD: Standard deviation; TMT: Trail Making Test. *p*-value: paired-samples *t*-tests (post-dose vs. pre-dose within each treatment arm). PRE: Baseline, 30 min: 30 min post-intervention, 3 h: 3 h post-intervention, 5 h: 5 h post-intervention.

Variables	Treatment	Mean	SD	% of Change from Baseline	*p*-Value
TMT-A PRE	Placebo	57.97	17.68	-	-
	ZYNS	61.84	23.69	-	-
TMT-A 30 min	Placebo	57.84	17.85	(−) 0.22	1.000
	ZYNS	55.51	18.33	(−) 10.24	0.009
TMT-A 3 h	Placebo	55.07	16.76	(−) 5.00	1.000
	ZYNS	43.48	15.13	(−) 29.69	<0.001
TMT-A 5 h	Placebo	54.99	16.58	(−) 5.14	0.863
	ZYNS	46.16	13.81	(−) 25.36	<0.001
TMT-B PRE	Placebo	58.95	19.91	-	-
	ZYNS	67.01	26.48	-	-
TMT-B 30 min	Placebo	56.44	21.16	(−) 4.26	1.000
	ZYNS	62.92	21.46	(−) 6.10	0.609
TMT-B 3 h	Placebo	55.55	21.41	(−) 5.78	0.891
	ZYNS	50.92	17.33	(−) 24.01	<0.001
TMT-B 5 h	Placebo	57.45	21.28	(−) 2.54	1.000
	ZYNS	52.88	17.76	(−) 21.10	<0.001
DSST PRE	Placebo	60.68	11.39	-	-
	ZYNS	59.13	14.51	-	-
DSST 30 min	Placebo	61.93	11.16	(+) 2.06	1.000
	ZYNS	64.73	11.98	(+) 9.47	<0.001
DSST 3 h	Placebo	62.85	11.19	(+) 3.58	0.623
	ZYNS	67.60	11.88	(+) 14.32	<0.001
DSST 5 h	Placebo	62.16	10.79	(+) 2.44	1.000
	ZYNS	69.20	11.44	(+) 17.03	<0.001
STROOP PRE	Placebo	61.07	7.69	-	-
W&C	ZYNS	58.39	5.20	-	-
STROOP 30 min	Placebo	59.59	4.73	(−) 2.42	0.418
W&C	ZYNS	59.67	3.83	(+) 2.20	0.687
STROOP 3 h	Placebo	60.80	4.44	(−) 0.45	1.000
W&C	ZYNS	59.86	3.78	(+) 2.53	0.397
STROOP 5 h	Placebo	59.38	4.38	(−) 2.77	0.254
W&C	ZYNS	61.13	5.41	(+) 4.69	0.007

**Table A2:** Descriptive statistics for emotional (POMS) variables and longitudinal change. Values represent raw means and standard deviation for each assessment. Percentage of change (%) indicates the mean individual variation from baseline to each timepoint. SD: Standard deviation; TMT: Trail Making Test. *p*-value: paired-samples *t*-tests (post-dose vs. pre-dose within each treatment arm). PRE: Baseline, 30 min: 30 min post-intervention, 3 h: 3 h post-intervention, 5 h: 5 h post-intervention.

Variables	Treatment	Mean	SD	% of Change from Baseline	*p*-Value
POMS PRE	Placebo	39.06	16.86	-	-
TMD	ZYNS	45.91	20.78	-	-
POMS 30 min	Placebo	38.92	19.07	(−) 0.35	1.000
TMD	ZYNS	37.58	16.85	(−) 18.14	<0.001
POMS 3 h	Placebo	35.61	17.09	(−) 8.82	0.168
TMD	ZYNS	34.75	16.72	(−) 24.31	<0.001
POMS 5 h	Placebo	35.68	18.86	(−) 8.64	0.316
TMD	ZYNS	34.85	17.38	(−) 24.08	<0.001
POMS PRE	Placebo	8.15	4.34	-	
Tension	ZYNS	8.67	4.87	-	
POMS 30 min	Placebo	8.56	5.43	(+) 5.02	1.000
Tension	ZYNS	7.65	4.12	(−) 11.80	0.191
POMS 3 h	Placebo	7.38	4.27	(−) 9.48	0.632
Tension	ZYNS	6.00	3.18	(−) 30.80	<0.001
POMS 5 h	Placebo	7.03	4.13	(−) 13.67	0.146
Tension	ZYNS	6.51	3.84	(−) 24.90	<0.001
POMS PRE	Placebo	11.77	5.58	-	-
Depression	ZYNS	14.25	7.41	-	-
POMS 30 min	Placebo	11.47	6.57	(−) 2.61	1.000
Depression	ZYNS	12.10	7.07	(−) 15.07	<0.001
POMS 3 h	Placebo	11.11	6.35	(−) 5.60	1.000
Depression	ZYNS	10.81	6.44	(−) 24.16	<0.001
POMS 5 h	Placebo	11.34	6.35	(−) 3.67	1.000
Depression	ZYNS	10.99	6.38	(−) 22.89	<0.001
POMS PRE	Placebo	9.42	4.93	-	-
Fatigue	ZYNS	11.61	5.39	-	-
POMS 30 min	Placebo	9.39	5.03	(−) 0.36	1.000
Fatigue	ZYNS	9.36	5.31	(−) 19.37	<0.001
POMS 3 h	Placebo	9.09	4.84	(−) 3.50	1.000
Fatigue	ZYNS	8.55	4.83	(−) 26.42	<0.001
POMS 5 h	Placebo	9.49	5.29	(+) 0.72	1.000
Fatigue	ZYNS	8.56	4.81	(−) 26.32	<0.001
POMS PRE	Placebo	8.08	3.88	-	-
Confusion	ZYNS	9.02	4.63	-	-
POMS 30 min	Placebo	7.80	4.53	(−) 3.52	1.000
Confusion	ZYNS	8.38	4.63	(−) 7.18	0.469
POMS 3 h	Placebo	7.08	4.25	(−) 12.38	0.138
Confusion	ZYNS	7.28	4.81	(−) 19.27	<0.001
POMS 5 h	Placebo	6.74	4.18	(−) 16.60	0.01
Confusion	ZYNS	6.94	3.95	(−) 23.05	<0.001
POMS PRE	Placebo	12.00	5.32	-	-
Anger	ZYNS	14.05	5.84	-	-
POMS 30 min	Placebo	11.78	5.71	(−) 1.80	1.000
Anger	ZYNS	13.28	5.69	(−) 5.42	0.366
POMS 3 h	Placebo	11.06	5.18	(−) 7.86	0.307
Anger	ZYNS	13.25	4.71	(−) 5.66	0.597
POMS 5 h	Placebo	11.09	6.04	(−) 7.58	0.484
Anger	ZYNS	13.17	5.16	(−) 6.23	0.556
POMS PRE	Placebo	10.36	5.22	-	-
Vigor	ZYNS	11.69	5.04	-	-
POMS 30 min	Placebo	10.07	5.26	(−) 2.85	1.000
Vigor	ZYNS	11.86	5.78	(+) 1.46	1.000
POMS 3 h	Placebo	10.10	5.38	(−) 2.52	1.000
Vigor	ZYNS	11.14	4.91	(−) 4.76	1.000
POMS 5 h	Placebo	10.01	5.19	(−) 3.40	1.000
Vigor	ZYNS	11.32	5.05	(−) 3.21	1.000

**Table A3:** Standardized effect sizes (Cohen’s d) for the treatment contrast at each timepoint were derived from the baseline-adjusted mean difference divided by the pooled baseline standard deviation. Values are interpreted using Cohen’s benchmarks (0.2 small, 0.5 medium, 0.8 large).

Endpoint	30 min	3 h	5 h
TMT-A	−0.188	−0.631	−0.499
TMT-B	+0.178	−0.296	−0.294
DSST	+0.246	+0.396	+0.572
Stroop PC	+0.038	−0.116	+0.292
Stroop T	−0.074	−0.152	+0.093
POMS TMD	−0.233	−0.208	−0.206
POMS Tension	−0.230	−0.332	−0.145
POMS Depression	−0.069	−0.212	−0.220
POMS Anger	+0.082	+0.206	+0.186
POMS Fatigue	−0.209	−0.310	−0.385
POMS Vigor	+0.204	+0.092	+0.144
POMS Confusion	−0.117	−0.070	−0.047

**Table A4:** Crossover-specific effects for each endpoint: sequence effect (Day 1 pre-dose baseline compared between sequences PZ and ZP, independent-samples *t*-tests), period effect (Period 1 vs. Period 2pre-dose baseline compared within subjects, paired-samples *t*-tests), and carryover effect (Day 7 pre-dose baseline compared between sequences, independent-samples *t*-tests).

Endpoint	Sequence t	Sequence p	Period t	Period p	Carryover t	Carryover p
TMT-A	−2.08	0.041	+7.52	<0.001	−0.31	0.760
TMT-B	−1.73	0.088	+7.61	<0.001	+2.25	0.027
DSST	−0.51	0.610	+0.76	0.448	−1.91	0.059
Stroop PC	+1.99	0.049	−1.08	0.282	−1.86	0.066
Stroop T	+1.65	0.103	−0.93	0.355	−1.54	0.128
POMS TMD	−2.09	0.039	+7.03	<0.001	+1.50	0.138
POMS Tension	−0.83	0.407	+6.75	<0.001	+0.24	0.809
POMS Depression	−2.33	0.022	+5.82	<0.001	+1.38	0.172
POMS Anger	−1.88	0.063	+5.24	<0.001	+1.70	0.092
POMS Fatigue	−2.93	0.004	+4.95	<0.001	+1.11	0.269
POMS Vigor	−1.57	0.121	+5.10	<0.001	+0.96	0.339
POMS Confusion	−0.47	0.637	+5.56	<0.001	+1.68	0.096
