# Supplementary material for: Cognitive and Mood Effects of a Soluble Mango Leaf Extract (Zynamite® S): A Randomized, Double-Blind, Placebo-Controlled, Crossover Replication Trial
Source: Pharmaceuticals (Basel). 2026 Jul 18;19(7):1112. doi: 10.3390/ph19071112 (PMC13414551; doi:10.3390/ph19071112)

Table S1. Phytochemical characterization of Zynamite® S (batch ZYN60S24).

Component / Parameter	Content (%)	Method
**Main xanthones**		
**Mangiferin (Standardized)**	60.0	HPLC-PDA
**Other Phytoconstituents**		
**Polyphenols & Glycosides**	12.0*	Folin-Ca. (Spectrophotometric Method)
**Polysaccharides**	6.0 – 20.0	Phenol-Sulphuric
**Safety & Purity Profile**		
**Fats & Lipids**	< 2.0	Gravimetric
**Urushiols**	Not Detected	LC-MS/MS
**Tertiary Alkaloids**	Not Detected	LC-MS/MS

Figure S1. HPLC-PDA chromatogram and inline UV spectrum of Zynamite® S (batch ZYN60S24). The dominant peak elutes at 6.30 min (mangiferin), displaying characteristic xanthone absorption maxima at λ_max = 256.6, 317.3, and 365.9 nm. Peak purity was confirmed by spectral homogeneity across the elution profile.

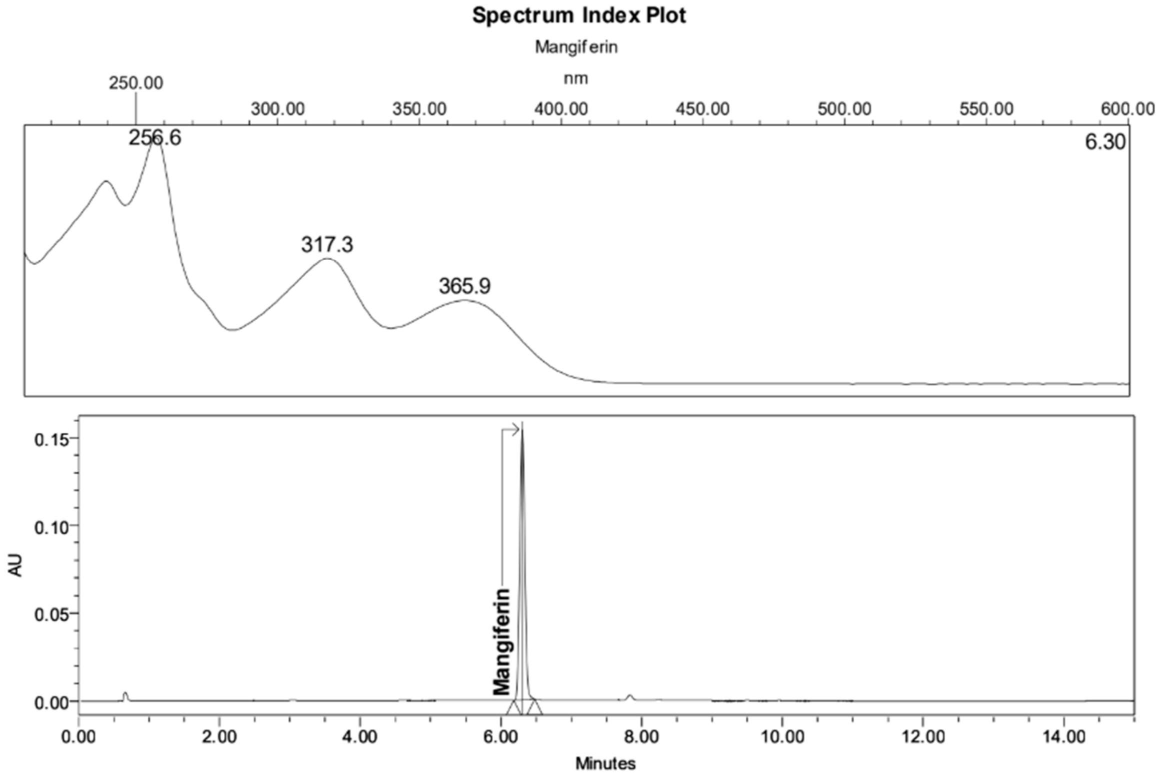

Figure S2. HPLC-MS/MS chromatographic and mass spectral characterization of Zynamite® S (batch ZYN60S24).

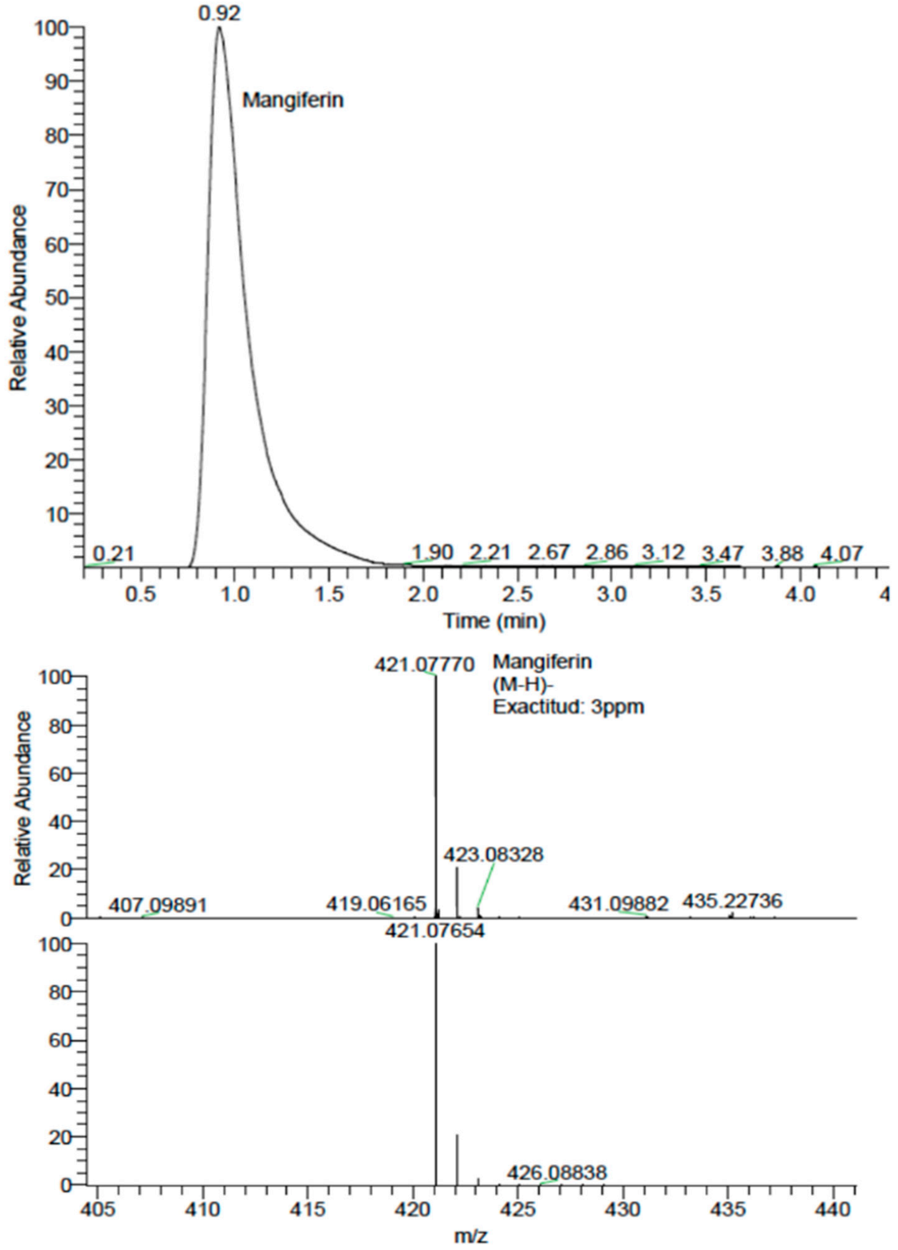

Supplement: Supplementary file 1 [file pharmaceuticals-19-01112-s001.zip › pharmaceuticals-4404625-supplementary.pdf]
